# The role of water, sanitation and hygiene interventions in reducing soil-transmitted helminths: interpreting the evidence and identifying next steps

**DOI:** 10.1186/s13071-019-3532-6

**Published:** 2019-05-28

**Authors:** Susana Vaz Nery, Amy J. Pickering, Ebba Abate, Abraham Asmare, Laura Barrett, Jade Benjamin-Chung, Donald A. P. Bundy, Thomas Clasen, Archie C. A. Clements, John M. Colford, Ayse Ercumen, Siobhan Crowley, Oliver Cumming, Matthew C. Freeman, Rashidul Haque, Birhan Mengistu, William E. Oswald, Rachel L. Pullan, Rita G. Oliveira, Katey Einterz Owen, Judd L. Walson, Ashrafedin Youya, Simon J. Brooker

**Affiliations:** 1 Kirby Institute, University of New South Wales, Sydney, Australia; 2 Department of Civil and Environmental Engineering, Tufts University, Medford, USA; 3 Ethiopian Public Health Institute, Addis Ababa, Ethiopia; 4 World Vision Ethiopia, Addis Ababa, Ethiopia; 5 Children’s Investment Fund Foundation, London, UK; 6 Division of Epidemiology and Biostatistics, School of Public Health, University of California, Berkeley, CA USA; 7 Faculty of Infectious and Tropical Diseases, London School of Hygiene & Tropical Medicine, London, UK; 8 Department of Environmental Health, Rollins School of Public Health, Emory University, Atlanta, USA; 9 Faculty of Health Sciences, Curtin University, Perth, Australia; 10 Department of Forestry and Environmental Resources, North Carolina State University, Raleigh, NC USA; 11 International Centre for Diarrheal Disease Research, Bangladesh, Dhaka, Bangladesh; 12 Neglected Tropical Diseases, Federal Ministry of Health, Addis Ababa, Ethiopia; 13 Global Health, Bill & Melinda Gates Foundation, Seattle, USA; 14 Department of Global Health, University of Washington, Seattle, USA; 15 Environmental Health, Federal Ministry of Health, Addis Ababa, Ethiopia

**Keywords:** Soil-transmitted helminths, Water, Sanitation, Hygiene, WASH

## Abstract

The transmission soil transmitted helminths (STH) occurs *via* ingestion of or contact with infective stages present in soil contaminated with human faeces. It follows therefore that efforts to reduce faecal contamination of the environment should help to reduce risk of parasite exposure and improvements in water, sanitation and hygiene (WASH) are seen as essential for the long-term, sustainable control of STH. However, the link between WASH and STH is not always supported by the available evidence from randomised controlled trials, which report mixed effects of WASH intervention on infection risk. This review critically summarises the available trial evidence and offers an interpretation of the observed heterogeneity in findings. The review also discusses the implications of findings for control programmes and highlights three main issues which merit further consideration: intervention design, exposure assessment, and intervention fidelity assessment.

## Background

Soil-transmitted helminths (STH) are a group of intestinal nematodes that include *Ascaris lumbricoides*, *Trichuris trichiura*, and the hookworm species, *Necator americanus*, *Ancylostoma duodenale* and *An. ceylanicum*. These species are some of the most common infections among humans, affecting over 1.5 billion individuals globally [1]. Infection occurs through accidental ingestion of eggs of *A. lumbricoides *and *T. trichiura* (and occasionally *A. duodenale*) or larval penetration of the skin by hookworm larvae present in contaminated soil [2]. In recent decades, the burden of STH has declined markedly: the 2016 Global Burden of Disease study estimated there was a 43–78% (depending on STH species) reduction in disability adjusted life years caused by STH between 1990 and 2016 [3]. These reductions likely reflect the direct impact of a scale-up in school- or community-based deworming programmes [4] as well as increased access to self-treatment.

It is also likely that economic development and increased access to improved water, sanitation and hygiene (WASH) infrastructure and services have contributed to the reduction in the STH disease burden, by reducing exposure to STH infective stages in the environment. Yet today too many people still lack access to basic WASH services, including 4.5 billion people without access to safely managed sanitation, 844 million without access to a basic water service, and 892 million people still practicing open defecation [5]. Addressing such inequalities has the potential to further reduce the burden of STH and potentially interrupt transmission.

Recent years have seen increased coordination and collaboration between the WASH and neglected tropical diseases sectors [6, 7]. However, key policy questions remain on the role of WASH in STH control, including: (i) whether improved access to WASH is an essential adjunct to deworming in order to control and eliminate STH; (ii) what are the appropriate WASH interventions and behaviours to achieve these goals; and (iii) how to best deliver those improvements. In this viewpoint, we report on an expert meeting convened by the Bill & Melinda Gates Foundation and the Children’s Investment Fund Foundation in London, January 29, 2018. The available evidence on the impact of WASH interventions on STH infection is reviewed and the main discussion from the meeting is presented.

## The current evidence

The potential for WASH to reduce STH transmission is supported by observational studies that report lower risk of STH infection associated with improved WASH access and practices. A 2014 systematic review and meta-analysis found that improved WASH, including piped water access, access to sanitation facilities, wearing shoes, and handwashing with soap, were associated with a 33–70% lower odds of STH infection [8]. A separate systematic review on the impact of sanitation found that sanitation was associated with 27% lower odds of *A. lumbricoides* infection, 20% lower odds of *T. trichiura* and 35% lower odds of hookworm infection [9].

Whilst observational studies can be useful and relatively quick and low-cost to conduct, they are vulnerable to systematic error or bias, including confounding. The strongest source of evidence to evaluate WASH interventions are cluster randomised controlled trials (RCTs), where interventions are randomised by groups of individuals, such as schools or communities. Cluster RCTs have their own limitations however, as they often focus on internal validity to assess a specific intervention within a specific context, sometimes at the detriment of external validity (i.e. generalisability). Table 1 summarises the designs and results of published cluster RCTs reporting the impact of school- and community-based WASH interventions on STH infections. Trials were identified by meeting participants who conducted many of the trials and had extensive knowledge of the field. The selection was supplemented by PubMed searches using key WASH and STH medical subject headings. The included trials have a variety of designs and outcomes, resulting in differing degrees of rigor, including the potential for contamination between arms, and this can hinder comparability across studies. However, some general results do emerge from the trial findings.

**Table 1:** Summary of cluster-randomised trial and non-randomised cluster intervention studies evaluating the effect of WASH interventions on soil-transmitted helminth infections

Setting	Design	Follow-up duration	Intervention and control	STH outcomes	Impact on STH
School-based trials					
Peru, Belén [11]	Pair-matched cluster-randomized controlled trial in 18 schools	4 months	*Intervention*: deworming followed by hygiene education which was supported by fortnightly support visits *Control*: deworming alone	STH infection; knowledge about STH among grade 5 school children (~12 years of age)	Intensity of *A. lumbricoides* 58% lower in the intervention arm but no impact on intensity of *T. trichiura* or hookworm; no impact on prevalence of any STH
China, Hunan Province [10]	Single-blind, unmatched, cluster-randomized trial in 38 urban schools	1 school year	*Intervention*: Behavior change intervention, including a cartoon video *Control*: display of a health-education poster	*A. lumbricoides* and *T. trichiura* infection; knowledge about STH; hand-washing behavior (hookworm not present)	50% reduction in STH infection
Kenya, Nyanza Province [12]	Cluster-randomized trial in 40 schools	10 months	*Intervention*: deworming followed by hygiene promotion, water treatment technology and supplies, latrine construction and hand washing and drinking water storage containers *Control*: deworming alone	STH reinfection among school children	44% reduction on *A. lumbricoides* reinfection, but no impact on other STH species
Community-based trials					
Northern Ethiopia [24]	Individual 2 × 2 factorial clustered randomized in 216 households	6 months	*Intervention*: promotion of handwashing with (provided) soap and nail clipping	STH reinfection among children aged 6–15 years	Lower rates of STH reinfection among children receiving handwashing intervention (14 *vs* 29%) and nail clipping (17 *vs* 26%)
India, Odisha [13]	Cluster-randomized trial in 100 rural villages	30 months	*Intervention*: latrine promotion and construction, as part of a CLTS campaign	STH infection among children aged <5 years	No impact on STH infection
India, Madhya Pradesh [14]	Cluster-randomized trial in 80 rural villages	21 months	*Intervention*: subsidies for and promotion of household latrines, as part of a CLTS campaign	STH infection among children aged <2 years	No impact on STH infection
Kenya, Western Province [16]	Cluster-randomized trial in 1226 villages grouped into 702 clusters	24 months	*Intervention*: 6 interventions including water, sanitation, handwashing, nutrition, combined WASH, and combined WASH plus nutrition (WSHN) *Control*: active and passive controls (no deworming)	STH infection among children aged 2 years and an older child in each household (mainly *A. lumbricoides*)	*A. lumbricoides* infection prevalence was 18% lower in the water arm, 22% lower in the combined WASH arm, and 22% lower in the WSHN arm
Bangladesh [17]	Cluster-randomized trial in 5551 compounds grouped into 702 clusters	26 months	*Intervention*: 6 interventions including water, sanitation, handwashing, nutrition, combined WASH, and combined WASH plus nutrition (WSHN) *Control*: passive controls (no deworming by study)	STH infection among children aged 2 years and up to 2 older children in each household	Water intervention reduced hookworm by 31% but did not affect other STH. Sanitation reduced *T. trichiura* by 29%, had a similar borderline effect on hookworm and no effect on *A. lumbricoides*. Combined WASH reduced hookworm by 29% and WSHN by 33%
Timor-Leste, Manufahi [19]	Cluster-randomized trial in 18 rural villages	24 months after 1st deworming	*Intervention*: provision of water, CLTS based sanitation promotion and handwashing education, integrated with deworming of whole communities *Control*: deworming of whole communities	STH infection among entire communities	No additional impact of WASH interventions on STH infection compared to deworming alone
Non-randomised studies					
Côte d’Ivoire, south-eastern [20]	Non-randomized cluster intervention study in 9 villages	13 months	*Intervention*: CTS campaign and hygiene education plus two rounds of community-based deworming *Control*: two rounds of community-based deworming	STH infection among household members (mainly hookworm present)	No statistically significant impact on STH prevalence, potentially higher hookworm ERR in intervention

*Abbreviations*: CLTS, community-led total sanitation; ERR: egg reduction rate; STH: soil-transmitted helminth

School-based hand hygiene and sanitation interventions can reduce STH reinfection among school children in some settings, but the impact varies by species. For example, hygiene promotion reduced prevalence and intensity of *A. lumbricoides* and *T. trichiura* in a trial in China [10] and intensity of *A. lumbricoides* infections, but not other species, in Peru [11]. WASH was also found to have an impact on *A. lumbricoides* alone in a trial of school-based WASH interventions in Kenya [12]. By contrast, a comprehensive school WASH intervention in Laos PDR did not reduce STH among school-aged children or their parents or under 5 siblings (Chard AN & Freeman MC, unpublished data).

Of the available community trials, most have focused on evaluating sanitation interventions aiming to reduce open defaecation. Three trials in India reported mixed effects from sanitation interventions on STH infection. Trials in Madhya Pradesh and Odisha in India reported no protective effects of latrine construction campaigns on STH infections [13, 14]. In both cases, however, open defaecation was still widespread in intervention communities due to low coverage and limited use of latrines. In a more recent study using a non-randomised, matched-cohort study design to assess the effect of a combined household water connection and latrine programme in Odisha, investigators reported a 44% reduction in overall STH infections (mainly hookworm, the most prevalent species) (Reese H et al., unpublished data). In that case, latrine coverage and coverage and use of household water supply connections were relatively high (>85%) and the intervention had been in place for at least three years before the study was undertaken, whereas follow-up in other studies has been shorter.

Other community randomised trials in different settings have evaluated the impact of single and combined WASH interventions on STH as secondary outcomes. Two recent, large factorial RCTs in rural Kenya and Bangladesh, known collectively as WASH Benefits, used similar trial designs to evaluate the effect of WASH interventions, alone and in combination, on STH infections in children in each setting. In Kenya, the trial achieved moderate-to-high levels of WASH coverage and found a lower prevalence of *A. lumbricoides* in the arm that received chlorine water treatment and in the combined WASH arm [15, 16]. In Bangladesh, sustained high uptake of the WASH interventions was achieved, and the trial found reductions of *T. trichiura* prevalence in the sanitation arm and hookworm in the chlorine water treatment arm and the integrated WASH intervention arm [17]. It is important to note that STH infections were not the primary outcomes for which the trials were designed, and as such the trials were powered to detect only relatively large reductions in STH.

Further trials have evaluated the impact of mass drug administration (MDA) *versus* MDA combined with WASH. In the WASH for WORMS trial conducted in Timor-Leste, an integrated programme of WASH and community MDA was found to have no additional impact on STH infection compared to MDA alone [18, 19]. In contrast, a non-randomised intervention study in Côte d’Ivoire reported greater egg reduction rates of hookworm among communities that received a combined programme of community-led total sanitation (CLTS) and community-wide MDA compared to community-wide MDA alone [20].

## Interpretation of findings

The above evidence demonstrates mixed findings. Potential explanations for such heterogeneity include that WASH interventions assessed in trials to date were either not appropriate to their study settings, were too complex resulting in limited uptake, failed to reach sufficiently high levels of coverage in the study population, or failed to achieve correct, consistent and sustained use. For instance, sanitation facilities, even those deemed “safely managed” under international monitoring standards, may present risks of user exposure inside (e.g., from unclean squatting slabs) and to the community from open sewers and untreated faecal sludge. WASH interventions have to date struggled to achieve and sustain high levels of community coverage and use, partly due to inadequate behaviour change methods to consistently achieve desired WASH practices. It is noteworthy that even in settings where high coverage of several WASH interventions is achieved, as in the Bangladesh WASH Benefits trial, the impact of interventions can still be modest.

In addition, since all communities have some access to water, some level of sanitation coverage and use and practice some personal hygiene behaviours, it is challenging to know what level of WASH intervention coverage is required to interrupt a sufficient number of exposure pathways and, in doing so, prevent reinfection. It is possible that there is a minimal required level of coverage and use for WASH interventions to have an impact, but this threshold will undoubtedly vary by intervention type, background reinfection rates, ongoing deworming or other factors not yet well defined. It is also difficult to manage contamination between arms in WASH RCTs, where control communities might have some uptake of improved water, sanitation or hygiene practices, diluting the estimated effect of the intervention.

Interestingly, the impact of WASH interventions in some trials was greatest for *A. lumbricoides* compared to hookworm. This may reflect differences in transmission (ingestion versus transdermal) or the ability of *A. lumbricoides* eggs to survive in the soil for years while hookworm larvae survive for weeks to months [21, 22]. Alternatively, the species-specific effects may reflect underlying differences in epidemiology of infection, including age patterns, within the respective study populations.

Lastly, the impact of WASH interventions seems to vary according to the underlying level of STH infection, with impact greatest at lower levels of infection. A possible explanation for this observation is that at high levels of infection and environmental contamination, WASH interventions require a longer follow-up period to see effects—most trials have follow-up periods of 1–2 years. Impact may additionally depend on the presence and length of ongoing deworming programmes. We hypothesize that the impact of WASH may be greatest after multiple years of MDA, when STH prevalence has been reduced to low levels. Here, mathematical modelling provides useful insights: using an individual-based model, Coffeng et al. show that WASH interventions have negligible short-term impact on STH infections in the context of ongoing deworming programmes, especially community-wide deworming, but that they are essential to prevent rebound of infection once deworming is stopped [23]. The dynamic interaction between intervention effort (coverage and efficacy) and infection transmission intensity is recognized in the design of vaccination programmes, and in STH population dynamic theory around MDA, but has yet to be examined seriously in the context of WASH programming. Modelling of WASH is an area which deserves more attention, using available trial data to improve the robustness of model predictions.

## Implications for programmes and future research

The London meeting identified three main issues that merit further consideration: intervention design, exposure assessment, and intervention fidelity assessment.

First, there is a need to identify and evaluate context-specific, feasible complementary interventions to support deworming and WASH programmes. Some of these complementary interventions can be remarkably simple and affordable. For example, a trial in Ethiopia showed that combined fingernail clipping with a handwashing intervention reduced STH reinfection rates among school-aged children [24]. Shoe wearing, improved flooring, food hygiene (both at the household and food-system level), household hygiene and health promotion to reduce geophagia (intentional consumption of soil) are additional interventions not typically included in WASH programmes but may reduce exposure to STH infective stages. As an illustration, an observational study in rural Bangladesh found that finished flooring was associated with lower *Ascaris* infections among children [25], highlighting the potential impact of improved household flooring. In practical terms, formative research is first needed to identify, develop, and assess the feasibility of specific intervention packages to control STH. Additional RCTs would then be needed for understanding if there are additional benefits to integrating these complementary interventions within deworming and WASH programmes.

Secondly, future work needs to improve and incorporate assessments of STH exposure from the environment, ideally using molecular assays. Measuring exposure to STHs has not typically been integrated into WASH or MDA trials. When it is, it consists of indirect measures of exposure (by STH loads in the soil) in an attempt to understand if interventions are actually interrupting or reducing environmental transmission of STH. Persistent environmental reservoirs of STH eggs may not be reduced by all types of WASH interventions, and could prevent intensive MDA programmes from achieving STH elimination. Reinfection rates six months after MDA range from 57% to 94% for STH species [26]. There are limited data on STH infective stages in the environment, as well as uncertainty regarding how long helminth eggs typically stay viable in soil, food and water and on hands and surfaces outside of a laboratory setting. Moreover, few studies have quantified the abundance and distribution of helminth eggs in soil [27], drinking water, on hands, or on surfaces. Environmental surveillance, coupled with new molecular markers of STH [28], could provide valuable insight into how environmental STH reservoirs are affected by specific interventions. Future work will also need to address how to standardise measurement of STH infective stages in the environment across matrices [29]. Taking the long view, an effective method for measuring the density of helminth eggs in the environment might provide a low-cost, less intrusive alternative to measuring human infection as a means of screening communities, as is done by screening sewage for polio virus.

Thirdly, we must improve our reporting of both intervention fidelity and measurement of WASH outcomes (i.e., intervention uptake and usage). There is a need to more clearly report on intervention fidelity with the use of standardized measures and process evaluation to understand why a programme works, and enhance the external validity and comparability of the studies [30]. Applying implementation science frameworks, as are used in other public health disciplines, could support our understanding of how to replicate and scale successful interventions [31]. We also need better measures of WASH outcomes as intermediate measures on the causal chain between interventions and STH outcomes. Evidence from recent WASH trials suggest that even potentially effective interventions often fail to achieve health impacts because of poor delivery and uptake by the target population [13, 14, 19]. Most interventions consist of a combination of technology and behaviour change. For example, improved flooring may be readily embraced by a population, but still require cleaning in order to maximize protection against STH infection; handwashing with soap requires substantial behaviour change, but compliance can be improved by hand washing stations located near latrines and food preparation areas. Sanitation interventions often have a very high uptake initially but use of latrines decreases overtime potentially due to poor construction quality. It will be important to identify a set of harmonised outcomes to help comparison across studies. Future trials also need to carefully consider the behaviour change required to improve update and usage, including understanding beliefs that could hinder behaviour change.

## Conclusions

The mainstay of current STH control programmes is periodic, population-based deworming (MDA), which has been shown to be safe, scalable and cost-effective. These programmes have been shown to provide health benefits for the recipients and have demonstrated value for endemic communities today. But MDA alone has yet to be shown to provide a long-term solution: indeed, potential anthelmintic resistance, donor fatigue and other threats raise concerns about the long-term sustainability of deworming programmes alone. Historical experience in previously endemic countries, including Europe, the USA, Japan and South Korea, has shown that STH infections can be effectively and sustainably controlled in the long-term through environmental interventions. Moreover, improvements in WASH will continue to be a major policy objective beyond the goal of reducing STH, due to its wide-ranging impacts on other diseases, society and well-being. Clearly MDA needs to continue where STH infection remains high and where WASH interventions cannot have an immediate impact. What is now required is stronger evidence and policy guidance on the complementary role that WASH has for deworming programmes (especially in preventing reinfection), the specific WASH interventions that have the greatest impact on STH, the WASH coverage levels which are required to have an impact on STH, and when in a control programme cycle they should be emphasized.

## Data Availability

Not applicable.

## Abbreviations

MDA: mass drug administration
RCT: randomized controlled trial
STH: soil-transmitted helminths
WASH: water, sanitation and hygiene
